# SOLACE Spectrum: A Personality Assessment for Personal Growth in Therapy

**DOI:** 10.3390/bs15111473

**Published:** 2025-10-29

**Authors:** Sherry R. Rosenblad, Carlos Guerrero, Jodie Lockeby, Dirce Utrera

**Affiliations:** 1 Department of Clinical Counseling and Mental Health, Texas Tech University Health Sciences Center, 3601 4th Street, Lubbock, TX 79430, USA; 2 Department of Educational Psychology, Leadership, & Counseling, Texas Tech University, 3002 18th Street, Lubbock, TX 79409, USA

**Keywords:** personality, personality assessment, personality traits, personal growth, identity development, personality in therapy, factor analysis

## Abstract

Personality assessment has long been recognized as a valuable tool for understanding individual differences with implications for self-understanding and growth-related processes. Building on the development of the Personality Spectrum Analysis (PSA), the present study evaluated the SOLACE Spectrum, a revised and expanded measure designed to provide a reliable and accessible framework for understanding personality in therapeutic and relational contexts. Data were collected from 1021 adults through online administration, and exploratory factor analysis revealed six components: Stability, Optimism, Leadership, Achievement, Compassion, and Extroversion. The instrument demonstrated strong internal consistency (α = 0.91) and robust test–retest reliability (0.851–0.922), indicating stability over time. Findings support the SOLACE Spectrum as a psychometrically sound measure that can inform understanding of personality traits, relationship processes, and personal growth processes. Its application may assist professionals in therapy, counseling, and educational or organizational settings by providing descriptive feedback on personality dimensions, highlighting areas of strength, and identifying potential areas for reflection and personal insight.

## 1. Introduction

Understanding personality is central to psychological research and practice, as it shapes identity development, personal growth, and well-being across the lifespan. While personality theories trace back to antiquity, validated and reliable assessments emerged primarily in the 19th century and have continued to evolve in response to advances in psychology and clinical practice. These instruments not only provide insight into individual differences but also serve as tools for fostering self-awareness, guiding therapeutic interventions, and supporting growth in relational, academic, and career domains. This article examines prominent personality assessments with attention to their efficacy and applicability, with a particular focus on the SOLACE Spectrum, a framework designed to enhance understanding of personality in the service of personal development and well-being.

Personality plays a fundamental role in psychology, shaping how individuals develop their sense of self, pursue personal growth, and psychological health (Bagby et al., 2016; Bucher et al., 2019). Personality psychology examines enduring patterns of thought, emotion, and behavior, with attention to both stability and variability across contexts (American Psychological Association, 2018). Scholars have long debated definitions, but most agree that personality reflects the consistent ways individuals interact with the world and adapt to their environment (Allport, 1961; Boag, 2011; Eysenck, 1970). Trait theories, in particular, propose that central dimensions of personality can predict behavior, though these must be understood alongside situational and biological influences (Boag, 2011; Friedman & Schustack, 2003; Widiger et al., 2019). Biological temperament—such as tendencies toward introversion or extroversion, emotional reactivity, or impulsivity—further shapes personality expression (Eysenck, 1970). Taken together, research supports the view that personality is best understood as enduring yet flexible patterns of behavior and interaction that provide meaningful insight into human motivation.

Efforts to translate theory into practice have led to numerous personality assessments, each with distinct strengths and limitations. Rosenblad (2014) developed the Personality Spectrum Analysis (PSA) as a practical, reliable, and accessible tool for identifying major personality dimensions. Based on data from 800 participants, the PSA was written at approximately a third-grade reading level to maximize accessibility and demonstrated strong psychometric properties, including evidence of content and construct validity. Internal consistency reliability for the overall instrument was 0.82, with individual component reliabilities ranging from 0.74 to 0.88, indicating acceptable to high reliability. Factor analysis identified six personality components—Achievement, Leadership, Compassion, Stability, Socializing, and Optimism—each characterized by distinct clusters of traits. To enhance clarity and clinical application, the framework was later refined into the SOLACE Spectrum, an acronym underscoring the role of personality awareness in providing “solace,” or comfort, through greater self-understanding and improved relationships.

### 1.1. Current Research Applications of Personality Assessment

Personality assessment has proven valuable in current research of counseling and supervision contexts. In clinical practice, assessment results support diagnosis, strengthen therapeutic alliance, guide treatment selection, and foster client insight (Bucher et al., 2019; Costa, 1991; Delgadillo et al., 2020; Holtzman & Raskin, 1989; Kamphuis & Finn, 2018). Clients who understand their personality patterns are better able to recognize strengths and address challenges, while counselors can tailor interventions to individual needs (Costa & McCrae, 2008; Delgadillo et al., 2020; Samuel et al., 2018). For example, individuals lower in openness may benefit from directive approaches, while those higher in openness may respond more effectively to experiential methods (Costa, 1991; Samuel et al., 2018). Personality assessment is also applied in couples and family counseling, where greater awareness of personality similarities and differences helps reduce conflict, improve communication, and enhance relational satisfaction (Lampis et al., 2018; Sperry & Carlson, 2000). Research further indicates that personality traits shape financial behaviors, conflict patterns, and relational adjustment (Jeanfreau et al., 2018; Myers et al., 2003). In supervision, both supervisors and trainees benefit from awareness of personality dynamics, which can improve communication, reduce conflict, and help trainees manage the stress of clinical training (Lewis et al., 2022; Rosenblad, 2014).

Beyond therapy, personality traits play a central role in shaping adolescents’ identity development (Hua & Zhou, 2023; Normandin et al., 2023). In addition, personality assessments have been widely used in career counseling, where matching individuals with roles that align with their traits is linked to higher job and life satisfaction (Granello & Young, 2019; Sowumni, 2022). In these contexts, personality serves as both a predictor of vocational success and a guide to well-being (Hua & Zhou, 2023).

### 1.2. Overview of Contemporary Personality Assessment Tools

#### 1.2.1. Myers–Briggs Type Indicator (MBTI)

Several well-known assessments illustrate the field’s diversity. The Myers–Briggs Type Indicator (MBTI) was derived from Carl Jung’s (1921/1974) theory of psychological types, which posited that personality arises from the interaction between introversion and extraversion and four mental functions—sensing, intuition, thinking, and feeling—later expanded by Briggs and Myers to include judgment and perception (Pearman & Albritton, 1997). The Myers–Briggs Type Indicator (MBTI) categorizes individuals into 16 types, with results presented through four-letter codes that can be difficult for clients to recall and apply without professional interpretation. While the MBTI has a strong research base and is easy to administer, it is relatively costly, and its deeper interpretive materials are dispersed across multiple sources, making it less accessible for everyday use (Myers et al., 2003).

#### 1.2.2. Big Five and NEO PI-R

The Big Five and NEO PI-R are grounded in trait theory and the lexical hypothesis, suggesting that enduring traits can be empirically identified through language and observed behavior (Costa & McCrae, 2008; Friedman & Schustack, 2003). These provide robust trait measures with high reliability, though their multidimensional scoring often requires the client to remember and interpret multiple domain and facet scores, which can limit their direct application to relationships or personal growth (Granello & Young, 2019). Furthermore, while early domains have shown strong reliability, the additional two domains of the NEO PI-R have required further validation (Piedmont, 2006).

#### 1.2.3. 16pf

The Sixteen Personality Factor Questionnaire (16pf) emerged from Raymond Cattell’s factor-analytic model of personality, which identified clusters of correlated behaviors representing underlying dimensions of personality (H. E. Cattell & Mead, 2008; R. B. Cattell & Schuerger, 2003). Despite its comprehensive nature, the 16PF’s presentation of multiple global and primary factors may overwhelm clients who struggle to recall or apply so many dimensions in daily life. Reliability coefficients for the 16PF range from questionable to good (0.60–0.89; A. Field, 2009), and while test–retest stability is stronger, it remains a relatively expensive and complex assessment to interpret.

#### 1.2.4. StrengthsFinder or CliftonStrengths

The StrengthsFinder (now CliftonStrengths) draws from positive psychology and talent theory, emphasizing innate patterns of thought, feeling, and behavior that can be developed into strengths. StrengthsFinder identifies individual talents and work styles but is framed more narrowly around occupational strengths making it less suitable for therapeutic exploration of interpersonal or emotional dynamics (Rath & Clifton, 2017).

#### 1.2.5. Enneagram

The Enneagram integrates insights from ancient character typologies and modern psychodynamic theory, describing nine core types organized around motivational and defense patterns. Although it offers an intuitive typology widely recognized in popular culture, empirical support for its construct validity remains mixed (Newgent et al., 2017). In addition, the Enneagram’s system of labeling types by number can make it difficult for individuals to recall what each number represents—both for themselves and for others they are in relationship with—limiting its practical application in improving relational understanding and communication.

#### 1.2.6. Personality Spectrum Analysis (PSA)

Finally, the Personality Spectrum Analysis (PSA) and subsequent SOLACE Spectrum are rooted in Adlerian theory, emphasizing social interest, relational functioning, and the individual’s unique style of life (Adler, 1956; Watts, 2012). The PSA operationalized these ideas into a practical typology to promote self-understanding and relational health, with the SOLACE Spectrum further refining the model for contemporary therapeutic and developmental use (Rosenblad, 2014).

While each assessment contributes valuable insight, limitations remain in cost, accessibility, and their usability in everyday contexts and relationships. Many popular instruments are either too expensive for routine use, too complex for clients to internalize, or too narrowly focused to address both intrapersonal and interpersonal functioning. The SOLACE Spectrum was developed to bridge these gaps—providing a theoretically grounded, psychometrically sound, and easily interpretable tool that fosters self-awareness, relational growth, and psychological well-being. The present study aims to clarify the personality dimensions originally identified in the PSA and to verify the factor structure, validity, and reliability of the SOLACE Spectrum as a tool aimed at providing insights into personal growth, relational health, and psychological well-being.

## 2. Materials and Methods

### 2.1. Transparency and Openness

All data are publicly available on the Open Science Framework (OSF) at www.osf.io/cmvkn. The study design and analyses were not preregistered. Data were analyzed using IBM SPSS Statistics (Version 29). The study was approved by the Hardin-Simmons University Institutional Review Board (IRB) in compliance with the DFFS Regulations for the Protection of Human Subjects (45 CFR-46).

### 2.2. Participants

#### 2.2.1. Main Sample

A priori power considerations: Given the 90-item instrument and planned factorial analyses, we targeted a sample of approximately 450–600 participants. This target aligns with commonly cited recommendations of at least five participants per item for factor analysis (Comrey & Lee, 1992; MacCallum et al., 1999) and ensures adequate power and stability for confirmatory factor analysis of a six-factor model (Fabrigar & Wegener, 2012). Additionally, this sample size provides more than 80–90% power to detect medium-sized correlations (r ≈ 0.30; Cohen, 1988).

The final sample for the SOLACE Spectrum revised instrument comprised 1021 adults. Of these, 69.4% identified as female and 30.6% as male, compared to U.S. Census figures of 51% female and 49% male. In terms of race and ethnicity, 73.7% identified as White (U.S. average = 60.1%), 12.3% as Hispanic (U.S. = 18.5%), 5.1% as African American (U.S. = 13.4%), 3.4% as Asian American (U.S. = 5.9%), 0.7% as Native American (U.S. = 1.3%), 2.8% as Multiracial (U.S. = 2.8%), and 2% as Other (U.S. = 1.3%). Table 1 provides full demographic information.

#### 2.2.2. Test–Retest Subsample

A separate subsample of 138 participants from the main dataset completed the SOLACE Spectrum on two occasions to assess test–retest reliability. Test–retest reliability examines the stability of results across multiple administrations of the same instrument. Although a minimum of 50 participants is recommended (Fraenkel & Wallen, 2006), power analysis indicated that a sample of 22–28 would be sufficient to detect a correlation of 0.80 with α = 0.05 and a medium effect size (Stevens, 2009). Thus, the sample of 138 participants provided power exceeding 0.94.

Participants self-reported the number of prior completions and the time elapsed since the previous completion. Only matched-pairs were retained for analysis to ensure data integrity. Intervals between completions ranged from 1 week to 74 months (M = 6 months). Although the range was broad, prior research supports the relative stability of personality traits across extended time periods, allowing for long-term test–retest comparisons (Costa & McCrae, 2008). Data were reviewed prior to analysis to verify accurate matching and ensure that no missing values were present in the paired dataset.

The test–retest sample was primarily between ages 20–29 (41.0%), female (69.1%), White (70.5%), single (66.9%), low-income (<$20,000/year, 62.6%), and having completed some college (29.5%). Detailed demographics are presented in Table 2.

### 2.3. Measures/Instruments

#### 2.3.1. Personality Spectrum Analysis Development

Rosenblad (2014) created the Personality Spectrum Analysis (PSA) as an accessible personality assessment. In its validation, the PSA was administered online to more than 1000 participants, of whom 800 were retained using stratified random sampling. The PSA demonstrated acceptable psychometric properties. Content and construct validity were established through literature, expert review, and factor analysis. Reliability was 0.82 overall, with component reliabilities ranging from 0.74 to 0.88, and a reading level of approximately 3.1 (Flesch-Kincaid). The PSA identified six personality components—Achievement, Leadership, Compassion, Stability, Socializing, and Optimism.

#### 2.3.2. SOLACE Spectrum

The SOLACE Spectrum refines and expands the PSA, retaining the six dimensions—Stability, Optimism, Leadership, Achievement, Compassion, and Extroversion—with revised labels and test items for greater clarity and applicability. The ‘Socializing’ dimension was renamed ‘Extroversion’ so that each label would begin with a different letter, forming the word ‘SOLACE’—symbolizing the comfort gained from better understanding yourself. Development of the SOLACE Spectrum followed standard test construction practices (Sheperis et al., 2020). A table of specifications was created to align content areas with the PSA-derived components. PSA items with low correlation values were revised or replaced with new items supported by the literature. Subject matter experts rated each item on a 5-point scale (1 = *very poor* to 5 = *very good*) for how well it represented its designated personality type. At least 25 questions were created for each domain. The 15 highest-rated items per dimension, based on inter-rater agreement, were retained for the final instrument. The instrument includes 90 items on a 5-point Likert scale of (1) *almost never*, (2) *rarely*, (3) *sometimes*, (4) *often*, and (5) *almost always* (See Appendix A). The measure emphasizes Adlerian principles, including social interest, relational functioning, and individual style of life.

### 2.4. Procedure

The study employed a quantitative survey design to collect self-report data on personality traits. Participants were recruited through convenience sampling (e.g., university courses taught by the primary investigator, local career counseling services, conference presentations, social media, and email signature links). Snowball sampling also occurred as participants shared the instrument with peers and family, extending recruitment across states and internationally. Approximately half of the participants were recruited through convenience sampling, and half through snowball sampling. After consenting, participants completed the SOLACE questionnaire online. For test–retest reliability, responses from repeated completions were matched, anonymized, and stored securely. Outliers and invalid cases were later screened (e.g., via Mahalanobis distance).

### 2.5. Scoring

Two items were reverse scored: Item #49 (“I do not like to take risks”) was reversed so that higher values reflected greater risk-taking (“I like to take risks”), and Item #50 (“I do not get worried easily”) was reversed so that higher values indicated greater worry or emotional reactivity (“I get worried easily”).

Each of the six personality factor subscales was calculated by summing the corresponding items as follows:

- Stability: Items 1, 7, 13, 19, 25, 31, 37, 43, 49 (reversed), 55, 61, 67, 73, 79, 85;
- Optimism: Items 2, 8, 14, 20, 26, 32, 38, 44, 50 (reversed), 56, 62, 68, 74, 80, 86;
- Leadership: Items 3, 9, 15, 21, 27, 33, 39, 45, 51, 57, 63, 69, 75, 81, 87;
- Achievement: Items 4, 10, 16, 22, 28, 34, 40, 46, 52, 58, 64, 70, 76, 82, 88;
- Compassion: Items 5, 11, 17, 23, 29, 35, 41, 47, 53, 59, 65, 71, 77, 83, 89;
- Extroversion: Items 6, 12, 18, 24, 30, 36, 42, 48, 54, 60, 66, 72, 78, 84, 90.

Raw subscale totals were transformed into z-scores to enable comparisons across dimensions within individual participants. For each individual, the z-scores across all six scales were centered at zero, with scores above zero reflecting traits more dominant in behavioral expression and scores below zero reflecting relatively less expressed tendencies. An online scoring calculator is available at www.SOLACESpectrum.com, which provides automated scoring and immediate feedback for users.

### 2.6. Data Analysis

Factorability checks: Mahalanobis distance was computed, and cases exceeding χ^2^ = 137.21 were excluded. The Kaiser–Meyer–Olkin (KMO) = 0.92 and sample size (>500) indicated excellent adequacy (A. Field, 2009; Hutcheson & Sofroniou, 1999). Bartlett’s Test of Sphericity was significant (*p* < 0.001), supporting factorability. Reproduced correlations ranged from –0.003 to 0.655, and only 8% of residuals exceeded 0.05, well below the 50% threshold, confirming linearity assumptions.

Factor extraction: Exploratory factor analysis was conducted using Equamax rotation, an orthogonal method that balances the simplification of variable loadings (as in Varimax) with the simplification of factor structures (as in Quartimax). This approach was selected to retain uncorrelated factors while improving interpretability across the multidimensional personality structure of the SOLACE Spectrum. Equamax was preferred over other orthogonal methods (e.g., Varimax) for its ability to balance item and factor simplicity, and over oblique rotations (e.g., Promax) to maintain orthogonality for clearer factor interpretability and independent subscale scoring (Tabachnick & Fidell, 2019). Six components were extracted.

Reliability testing: Cronbach’s α for internal consistency. Intraclass correlation coefficients (ICCs) with 95% confidence intervals were computed using a mean rating (k = 2), absolute agreement, and a two-way mixed-effects model (Koo & Li, 2016). Bland–Altman analysis assessed agreement across administrations.

## 3. Results

### 3.1. Test–Retest Reliability

Test–retest reliability was examined using a subsample of 138 participants who completed the SOLACE Spectrum on two separate occasions. Temporal stability was assessed through intraclass correlation coefficients (ICCs) calculated between the initial and subsequent administrations. Analyses were conducted using a two-way mixed-effects model with absolute agreement to evaluate the consistency of scores across time points.

ICCs for all six SOLACE components ranged from good to excellent (see Table 3), demonstrating strong temporal stability. Specifically, ICCs ranged from 0.851 to 0.922 across dimensions, indicating good to excellent agreement according to established benchmarks (Koo & Li, 2016; Perinetti, 2018; Cicchetti, 1994).

To further evaluate agreement between test and retest administrations, Bland–Altman analyses were conducted. These analyses indicated minimal mean bias (<0.1) across all SOLACE components (Bland & Altman, 1986, 1999). Between 92.8 and 94.9% of score differences fell within confidence limits, demonstrating strong overall agreement. Although some proportional bias appeared in Extroversion, 93.5% of differences remained within the limits of agreement (see Table 4).

Together, these findings provide strong evidence of the temporal stability and reliability of the SOLACE Spectrum across both short- and long-term intervals, supporting its use as a consistent measure of personality attributes over time.

### 3.2. Factor Analysis

After excluding multivariate outliers, KMO and Bartlett tests confirmed sampling adequacy and factorability. Six factors were extracted, explaining 40.95% of the variance. Though moderate, this variance is consistent with typical findings in personality and social–behavioral research, where complex constructs and item heterogeneity often limit explained variance (Costello & Osborne, 2005; Fabrigar et al., 1999).

### 3.3. Internal Consistency

The overall SOLACE Spectrum demonstrated strong reliability, with Cronbach’s α = 0.91. Subscale reliabilities ranged from 0.72 to 0.88, indicating acceptable to high internal consistency across dimensions (Sheperis et al., 2020; see Table 5).

### 3.4. Factor Descriptions

Each component is described below (with number of items, α, and variance explained):

- Achievement (α = 0.88; 19 items; 8.52% variance): Characterized by initiative, decisiveness, productivity, and high standards; individuals are efficient problem-solvers but may become over-responsible.
- Compassion (α = 0.83; 17 items; 7.37% variance): Defined by generosity, loyalty, and service orientation; individuals gain self-worth through helping others. One item (reverse-coded) indicated attraction to highly emotional individuals.
- Optimism (α = 0.85; 16 items; 7.20% variance): Marked by positivity, calmness, and resilience; individuals maintain a relaxed, hopeful outlook even during crises.
- Extroversion (α = 0.88; 13 items; 7.12% variance): Associated with sociability, expressiveness, and energy in group settings; individuals thrive in social contexts and are often perceived as charming and likable.
- Leadership (α = 0.81; 16 items; 5.86% variance): Defined by risk-taking, perseverance, and authority-seeking; individuals exhibit a strong work ethic, strive for recognition, and prefer control in group settings.
- Stability (α = 0.72; 9 items; 4.89% variance): Characterized by caution, tradition, and a preference for predictability; individuals seek security and avoid uncertain or embarrassing situations.

Full item loadings and reliabilities are presented in Table 6. Factor loadings of 0.30 or higher are generally considered the minimum threshold for inclusion on a factor (A. P. Field, 2018; Tabachnick & Fidell, 2019). Loadings >0.40 are bolded. Although item 50 had a corrected item-total correlation just below the recommended 0.30, it was retained because it represented the highest loading for its factor, and its removal did not improve the reliability of the component. Therefore, it was retained to preserve theoretical coverage and scale consistency. Though the question is the opposite of worried, it is reverse-scored and labeled worried.

For complete factor loadings above 0.10 after Equamax rotation, see Appendix B. Because personality is conceptualized as a spectrum, some variables load substantially on two components; the highest loading was used to assign each trait to its primary personality type, though certain traits may be expressed across multiple types.

## 4. Discussion

The SOLACE Spectrum advances personality assessment by combining psychometric rigor with practical usability. Grounded in Adlerian principles of social interest, relational functioning, and individual style of life (Adler, 1956; Watts, 2012), the instrument operationalizes these principles into six dimensions—Stability, Optimism, Leadership, Achievement, Compassion, and Extroversion—translating theory into a usable framework for applied settings. The primary aim of SOLACE is to provide a theoretically grounded, empirically validated, and practically usable personality assessment that supports self-awareness, relational understanding, and personal growth.

This study extends prior work with the Personality Spectrum Analysis (PSA) by validating the SOLACE Spectrum in a substantially broader and more diverse participant base. While approximately 90% of participants were from Texas, respondents also represented multiple U.S. states and international locations, enhancing the generalizability of the findings. Psychometric evaluation confirmed a six-factor structure consistent with theoretical expectations, with each dimension demonstrating good to excellent internal consistency and strong test–retest reliability. These findings support the instrument’s construct validity and suggest that SOLACE reliably captures meaningful personality factors.

The accessible and descriptive nature of SOLACE allows users to identify personal strengths and areas for growth, apply insights in interpersonal interactions, and align behavior patterns with occupational preferences, potentially enhancing career satisfaction and engagement. Awareness of one’s own traits, as well as the traits of others, may foster empathy, tolerance, and understanding in relational contexts such as couples, families, and professional teams, which can contribute to improved communication and reduced relational tension. Although SOLACE provides descriptive feedback and supports reflection, its predictive validity regarding personal growth, self-awareness, relational satisfaction, or well-being has not yet been established. The instrument’s value lies in summarizing personality traits in an accessible format to inform counseling, supervision, and interpersonal understanding without overextending claims.

Several methodological limitations warrant consideration. The sample, though geographically diverse, was primarily composed of English-speaking participants from Texas, which may limit broader generalizability. Data were collected through self-report measures, introducing potential response bias. While exploratory factor analysis confirmed the six-factor structure, confirmatory analyses with independent samples are needed to evaluate stability and generalizability. Future research should examine predictive and discriminant validity across applied contexts, as well as potential cross-cultural validation and longitudinal outcomes.

Future studies may also explore the development of a shorter version of SOLACE that retains the core content of each dimension while improving ease of administration and accessibility for both researchers and practitioners. Although a shorter form may slightly reduce reliability, high correlations with the full instrument could ensure accurate measurement of the primary dimensions, supporting broader adoption in applied research, counseling, educational, and organizational settings.

## 5. Conclusions

The SOLACE Spectrum represents an accessible, evidence-based instrument that translates Adlerian theory into a practical framework for personality assessment. Results from this study confirm its reliability and validity across six dimensions, offering a robust tool for both research and applied contexts.

When used in counseling or organizational settings, the SOLACE Spectrum can support clients in identifying personal strengths, understanding relational dynamics, and making informed career choices. While the instrument shows promise for enhancing self-awareness, relational understanding, and vocational alignment, further research is needed to examine its predictive utility for personal growth, well-being, and applied outcomes. Overall, the SOLACE Spectrum contributes to the field by providing a theoretically informed, empirically validated approach to understanding personality in diverse contexts.

## Figures and Tables

**Table 1:** Participant Demographic Information (*N* = 1021).

Participants	*N*	%
Age		
18–19	313	30.7
20–29	348	34.1
30–39	119	11.7
40–49	94	9.2
50–59	86	8.4
60–69	50	4.9
70–79	7	0.7
80 and up	1	0.1
Missing	3	0.3
Gender		
Female	709	69.4
Male	312	30.6
Race/Ethnicity		
White	752	73.7
Hispanic or Latino	126	12.3
African American	52	5.1
Asian	35	3.4
Native American	7	0.7
Multi-racial	29	2.8
Other	20	2.0
Marital Status		
Single	607	59.5
Married	352	34.5
Separated	5	0.5
Divorced	48	4.7
Widowed	8	0.5
Income		
Less than $20,000	557	54.6
$20,001–50,000	240	23.5
$50,001–80,000	153	15.0
$80,001–110,000	46	4.5
Over $110,000	25	2.4
Level of Education		
Not completed high school	73	7.1
Completed high school	178	17.4
Some college	259	25.4
Associate’s Degree	55	5.4
Bachelor’s Degree	238	23.3
Graduate Degree	218	21.4

**Table 2:** Test–Retest Participant Demographic Information (*N* = 138).

Participants	*N*	%
Race/Ethnicity		
White	98	70.5
Hispanic or Latino	17	12.2
African American	12	8.6
Asian	5	3.6
Native American	0	0
Multi-racial	5	3.6
Other	2	1.4
Marital Status		
Single	93	66.9
Married	39	28.1
Separated	5	3.6
Divorced	1	0.7
Widowed	1	0.7
Income		
Less than $20,000	87	62.6
$20,001–50,000	29	20.9
$50,001–80,000	14	10.1
$80,001–110,000	5	3.6
Over $110,000	4	2.9

**Table 3:** Intraclass Correlations between the test and retest assessment.

Component	ICC	95% Confidence Interval		Agreement
		Lower	Upper	
Stability	0.881	0.832	0.915	Good to Excellent
Optimism	0.919	0.887	0.942	Good to Excellent
Leadership	0.883	0.837	0.917	Good to Excellent
Achievement	0.851	0.792	0.893	Good
Compassion	0.878	0.830	0.913	Good to Excellent
Extroversion	0.922	0.891	0.944	Good to Excellent

Note. ICC reliability determinants used were: values less than 0.5 were poor, values between 0.5 and 0.75 were considered fair, values between 0.75 and 0.9 were good, and values over 0.90 were considered excellent reliability (Koo & Li, 2016; Perinetti, 2018).

**Table 4:** Bland–Altman limits of agreement analysis.

Component	Difference Mean (SD)	95% Confidence Interval		% Outside the LoA Interval	% Within the LoA Interval
		Lower	Upper		
Stability	0.099 (0.53)	−0.932	1.130	5.1%	94.9%
Optimism	−0.056 (0.43)	−0.899	0.788	5.8%	94.2%
Leadership	−0.038 (0.50)	−1.015	0.939	5.8%	94.2%
Achievement	0.030 (0.51)	−0.979	1.039	7.2%	92.8%
Compassion	0.016 (0.48)	−0.926	0.958	7.2%	92.8%
Extroversion	−0.051 (0.51)	−1.060	0.957	6.5%	93.5%

**Table 5:** Component Cronbach’s α Reliability Coefficients.

Component	Cronbach’s α	Reliability Determinant
1	0.88	High
2	0.83	High
3	0.85	High
4	0.88	High
5	0.81	High
6	0.72	Acceptable

**Table 6:** Primary Component Loadings from the Rotated Factor Matrix.

Item	Variable	Component					
		1	2	3	4	5	6
9	Initiative	**0.690**					
15	Take-charge	**0.647**					
43	Productive in challenge	**0.628**					
10	Productive	**0.615**					
52	Efficient	**0.609**					
28	Accept challenge	**0.572**					
16	Problem-solve	**0.551**					
40	Goal-oriented	**0.510**					
34	Over-responsible	**0.488**					
58	Detail problem-solve	**0.473**					
3	Decisive	**0.466**					
76	Skilled	**0.458**					
64	Right way	**0.453**					
55	Reliable	**0.429**					
22	High standards	**0.419**					
39	Strong	**0.410**					
4	Charts	0.355					
27	Influential	0.354					
88	Not relaxed	0.327					
23	Concern for others		**0.757**				
11	Thoughtful		**0.735**				
35	Help others		**0.732**				
59	Sacrificial		**0.695**				
41	Considerate		**0.693**				
89	Generous		**0.608**				
29	Listen		**0.535**				
53	Serve		**0.525**				
77	Worth from helping		**0.513**				
5	Help by problem-solve		**0.494**				
71	Polite		**0.474**				
65	Emotional intelligence		**0.423**				
83	Accept others		0.397				
86	Don’t like emotions		−0.381				
19	No conflict		0.375				
17	Forgive		0.370				
47	Loyal		0.367				
44	Calm under pressure			**0.700**			
62	Easy-going			**0.678**			
68	Level-headed			**0.608**			
32	Clear under pressure			**0.591**			
14	Calm under pressure 2			**0.581**			
38	Not upset			**0.553**			
56	Relaxed			**0.547**			
61	Self-control			**0.515**			
31	Clear-headed			**0.512**			
74	Carefree			**0.509**			
26	Calm in crisis			**0.475**			
20	Optimistic 3			**0.452**			
8	Optimistic 2			0.362			
75	Future vision			0.356			
80	Not detailed			0.342			
2	Optimistic			0.332			
42	Social				**0.783**		
24	Time with others				**0.707**		
6	Make friends				**0.702**		
18	Tell others				**0.691**		
36	Extrovert				**0.668**		
12	Expressive				**0.629**		
66	Like groups				**0.627**		
30	Lots friends				**0.560**		
72	Center attention				**0.527**		
60	Overshare				**0.507**		
84	Charming				**0.475**		
48	Depend on others				**0.450**		
90	Work with others				**0.435**		
82	Recognition for work					**0.659**	
81	Outperform					**0.620**	
21	Want recognition					**0.595**	
45	Express strength					**0.585**	
87	Authority					**0.538**	
57	Want respect					**0.538**	
63	Want challenge					**0.462**	
51	Want it right way					**0.419**	
33	Want excitement					**0.419**	
69	Often right					0.390	
70	Perfectionist					0.388	
73	Self-sufficient					0.365	
49	Take risks					0.346	
78	Decide with heart					0.338	
54	Want acceptance					0.332	
67	No change					0.314	
25	Know what to expect						**0.674**
13	Want safety						**0.594**
7	Careful plan						**0.586**
1	Want facts/prepare						**0.532**
79	Want expectations						**0.511**
37	Avoid embarrassment						**0.506**
46	Facts to problem-solve						**0.485**
85	Traditional						0.378
50	Worried						0.285

Factor loadings greater than 0.30 are displayed only for the factor where the loading was the highest. Factor loadings greater than 0.40 are in boldface.

## Data Availability

All data are publicly available on the Open Science Framework (OSF) at www.osf.io/cmvkn. The study design and analyses were not preregistered.

## Abbreviations

The following abbreviations are used in this manuscript:

PSA	Personality Spectrum Analysis
SOLACE Spectrum	Stability, Optimism, Leadership, Achievement, Compassion, Extroversion acronym
IRB	Institutional Review Board
IBM SPSS Statistics	Statistical Package for the Social Sciences
OSF	Open Science Framework

## Appendix A

SOLACE Spectrum Instrument Questions (www.SOLACESpectrum.com)

1. I want others to give me the facts so I can be prepared.
2. I expect problems to work out all right in the end.
3. I act on ideas decisively.
4. I like to organize information into charts or graphs.
5. I find a way to solve problems that helps others.
6. I make friends easily.
7. I prefer to do things after careful planning.
8. I see the world as “the glass is half full.”
9. I take the first step when there is a job to be done.
10. I find ways to get things done well without wasting time.
11. I am thoughtful of other people, what they want or need.
12. I express myself openly.
13. I like to feel safe.
14. I am able to stay calm in challenging situations.
15. I am a take-charge person.
16. I try to find solutions to problems.
17. I would rather forgive someone than hold it against them.
18. I often let others know what is happening in my life.
19. I avoid fights with others because I do not like conflict.
20. I believe the future will turn out all right.
21. I want people to notice my hard work.
22. I have very high expectations of myself.
23. I am very concerned about the needs of others.
24. I like spending time with others.
25. I like to know what to expect.
26. In a crisis, I am calm until it is over.
27. I am able to influence the actions of others.
28. I accept challenges.
29. I am a good listener.
30. I have a wide range of friends.
31. I am able to think clearly and to make good decisions.
32. If there is a problem, I am able to think clearly until it is resolved.
33. I look for opportunities that are exciting to me.
34. I assume more than my fair share of responsibility for tasks.
35. I like to help others in times of need.
36. I have more energy after spending time with others.
37. I avoid doing things that will embarrass me.
38. It takes a lot to upset me.
39. I am aware that I am a strong person.
40. I set a goal and try to achieve it.
41. I consider other’s feelings when making a decision.
42. I am seen as someone who enjoys people and social settings.
43. I am able to get things done in challenging situations.
44. I remain calm under pressure when others are stressed.
45. I like to express my strength.
46. I want others to give me the facts so I can solve the problem.
47. I am loyal.
48. I depend on others for support and help.
49. I do not like to take risks.
50. I do not get worried easily.
51. I try to make others do things the way I think they should be done.
52. I am efficient and do not waste time.
53. I look for opportunities to be of service.
54. I want others to like me.
55. People can count on me to do what I say.
56. I am relaxed.
57. I want others to admire and respect me.
58. I am good at breaking down a problem into smaller parts to find an answer.
59. I am willing to sacrifice for others.
60. Sometimes I over-share about myself.
61. I have a lot of self-control.
62. I have an easy-going manner.
63. I want to have challenges so I am not bored.
64. I get things done the right way.
65. I can usually tell how others are feeling.
66. I feel more comfortable in groups than by myself.
67. I create an environment that is not easily changed.
68. I tend to be level-headed.
69. I am most often right.
70. I expect my own work to be very close to perfect.
71. I am polite.
72. I feel well-liked when I am the center of attention.
73. I want to solve my problems myself.
74. I tend to have a carefree attitude.
75. I have good ideas about the future.
76. I have the necessary ability or skills to complete my tasks or objectives.
77. I feel like I am worth something if I help others.
78. I sometimes make a decision because it feels right.
79. I would rather know if I am working alone or with a group and be prepared.
80. I feel like the details will take care of themselves.
81. I try to outperform others even if I am the only one who notices.
82. I try to get recognition through doing good work.
83. I accept other people’s choices without trying to change them.
84. Others find me charming.
85. It is important to me to observe the same traditions every year.
86. I prefer not to be around people who are overly emotional.
87. I want to have authority.
88. I do not take time to rest or relax.
89. I am generous.
90. I work well with others.

## Appendix B

**Table A1:** Component Loadings for the Rotated Factor Matrix Over 0.10.

Item	Variable	Component					
		1	2	3	4	5	6
9	Initiative	**0.690**	0.135	0.146	0.143		
15	Take-charge	**0.647**			0.306	0.285	
43	Productive in challenge	**0.628**		0.340		0.213	
10	Productive	**0.615**		0.156			0.181
52	Efficient	**0.609**		0.169			0.223
28	Accept challenge	**0.572**		0.258		0.324	−0.221
16	Problem-solve	**0.551**	0.252	0.178		0.197	
40	Goal-oriented	**0.510**	0.185	0.190		0.247	0.144
34	Over-responsible	**0.488**	0.296			0.175	0.117
58	Detail problem-solve	**0.473**		0.321		0.151	0.139
3	Decisive	**0.466**		0.233			0.146
76	Skilled	**0.458**	0.175	0.301		0.199	0.146
64	Right way	**0.453**		0.155		0.210	0.256
55	Reliable	**0.429**	0.236	0.296			0.192
22	High standards	**0.419**	0.167			0.254	0.183
39	Strong	**0.410**		0.402	0.193	0.250	
4	Charts	0.355					0.283
27	Influential	0.354		0.219	0.350	0.319	−0.121
88	Not relaxed	0.327		−0.318		0.108	
23	Concern for others		**0.757**		0.169		0.109
11	Thoughtful		**0.735**		0.165	−0.140	0.101
35	Help others	0.153	**0.732**		0.181		
59	Sacrificial	0.142	**0.695**				
41	Considerate		**0.693**		0.141	−0.100	0.193
89	Generous		**0.608**	0.154	0.145		
29	Listen		**0.535**	0.230			
53	Serve	0.334	**0.525**		0.149	0.120	
77	Worth from helping		**0.513**		0.140	0.317	0.157
5	Help by problem-solve	0.395	**0.494**		0.145		
71	Polite		**0.474**	0.356			0.176
65	Emotional intelligence		**0.423**		0.261		
83	Accept others	−0.149	0.397	0.275			
86	Don’t like emotions	0.105	−0.381	0.224	−0.117	0.145	0.105
19	No conflict	−0.258	0.375	0.124		−0.100	0.304
17	Forgive		0.370	0.368	0.152	−0.202	
47	Loyal		0.367	0.178			0.185
44	Calm under pressure			**0.700**			
62	Easy-going	−0.233	0.238	**0.678**			−0.118
68	Level-headed	0.226	0.113	**0.608**			0.191
32	Clear under pressure	0.203		**0.591**			
14	Calm under pressure 2	0.313		**0.581**			−0.181
38	Not upset	0.238		**0.553**			−0.190
56	Relaxed	0.302		**0.547**		0.124	
61	Self-control	0.249	0.131	**0.515**	−0.142		
31	Clear-headed	0.490		**0.512**			0.170
74	Carefree	−0.287	0.101	**0.509**	0.100	0.165	−0.214
26	Calm in crisis	0.341		**0.475**			−0.123
20	Optimistic 3		0.263	**0.452**	0.233		
8	Optimistic 2	0.133	0.177	0.362	0.298	−0.134	
75	Future vision	0.220	0.220	0.356	0.162	0.290	
80	Not detailed	−0.309		0.342	0.194	0.181	−0.181
2	Optimistic		0.121	0.332	0.166		0.128
42	Social		0.118		**0.783**	0.136	−0.135
24	Time with others		0.258		**0.707**		
6	Make friends	0.118	0.142	0.152	**0.702**		−0.142
18	Tell others				**0.691**		0.197
36	Extrovert		0.126		**0.668**	0.159	
12	Expressive	0.220			**0.629**		
66	Like groups	−0.122	0.108		**0.627**	0.145	−0.109
30	Lots friends		0.187	0.136	**0.560**		−0.154
72	Center attention	−0.140			**0.527**	0.470	
60	Overshare			−0.219	**0.507**	0.138	0.176
84	Charming		0.150	0.249	**0.475**	0.303	−0.149
48	Depend on others	−0.137	0.121	−0.130	**0.450**		0.306
90	Work with others		0.391	0.284	**0.435**		
82	Recognition for work			−0.147		**0.659**	
81	Outperform	0.137				**0.620**	
21	Want recognition			−0.112	0.110	**0.595**	0.285
45	Express strength		−0.127		0.243	**0.585**	0.154
87	Authority	0.302	−0.203		0.173	**0.538**	
57	Want respect				0.280	**0.538**	0.261
63	Want challenge	0.388		0.177		**0.462**	−0.224
51	Want it right way	0.218	−0.268	−0.148	0.191	**0.419**	0.228
33	Want excitement	0.146	0.111	0.197	0.219	**0.419**	−0.133
69	Often right	0.323	−0.212	0.188		0.390	0.140
70	Perfectionist	0.384			−0.192	0.388	0.275
73	Self-sufficient	0.262		0.162	−0.320	0.365	
49	Take risks			0.204	0.106	0.346	−0.342
78	Decide with heart		0.303	0.151	0.278	0.338	
54	Want acceptance	−0.261	0.134		0.320	0.332	0.296
67	No change					0.314	0.246
25	Know what to expect	0.147				0.122	**0.674**
13	Want safety		0.274				**0.594**
7	Careful plan	0.315			−0.119		**0.586**
1	Want facts/prepare	0.250					**0.532**
79	Want expectations	0.109			−0.123	0.207	**0.511**
37	Avoid embarrassment	−0.154			−0.250		**0.506**
46	Facts to problem-solve	0.338				0.254	**0.485**
85	Traditional					0.141	0.378
50	Worried	−0.144	0.182	−0.258			0.285

Factor loadings > 0.10 are shown; loadings > 0.40 are in bold. Personality traits may load on multiple factors, reflecting the spectrum of personality, but each trait is assigned to the factor with its highest loading.

## References

[B1-behavsci-15-01473] Adler A., Ansbacher H. L., Ansbacher R. R. (1956). The individual psychology of Alfred Adler: A systematic presentation in selections from his writings.

[B2-behavsci-15-01473] Allport G. W. (1961). Pattern and growth in personality.

[B3-behavsci-15-01473] American Psychological Association (2018). Dictionary of psychology.

[B4-behavsci-15-01473] Bagby R., Gralnick N., Al-Dajani N., Uliaszek A. A. (2016). The role of the five-factor model in personality assessment and treatment planning. Clinical Psychology: Science and Practice.

[B5-behavsci-15-01473] Bland J. M., Altman D. G. (1986). Statistical method for assessing agreement between two methods of clinical measurement. The Lancet.

[B6-behavsci-15-01473] Bland J. M., Altman D. G. (1999). Measuring agreement in method comparison studies. Statistical Methods in Medical Research.

[B7-behavsci-15-01473] Boag S. (2011). Explanation in personality psychology: “Verbal magic” and the five-factor model. Philosophical Psychology.

[B8-behavsci-15-01473] Bucher M. A., Suzuki T., Samuel D. B. (2019). A meta-analytic review of personality traits and their associations with mental health treatment outcomes. Clinical Psychology Review.

[B9-behavsci-15-01473] Cattell H. E., Mead A. D., Boyle G. J., Matthews G., Saklofske D. H. (2008). The sixteen personality factor questionnaire (16PF). The sage handbook of personality theory and assessment.

[B10-behavsci-15-01473] Cattell R. B., Schuerger J. M. (2003). Essentials of 16PF assessment.

[B11-behavsci-15-01473] Cicchetti D. V. (1994). Guidelines, criteria, and rules of thumb for evaluating normed and standardized assessment instruments in psychology. Psychological Assessment.

[B12-behavsci-15-01473] Cohen J. (1988). Statistical power analysis for the behavioral sciences.

[B13-behavsci-15-01473] Comrey A. L., Lee H. B. (1992). A first course in factor analysis.

[B14-behavsci-15-01473] Costa P. T. (1991). Clinical use of the five-factor model: An introduction. Journal of Personality Assessment.

[B15-behavsci-15-01473] Costa P. T., McCrae R. R., Boyle G. J., Matthews G., Saklofske D. H. (2008). The revised NEO personality inventory (NEO PI-R). The sage handbook of personality theory and assessment.

[B16-behavsci-15-01473] Costello A. B., Osborne J. (2005). Best practices in exploratory factor analysis: Four recommendations for getting the most from your analysis. Practical Assessment, Research, and Evaluation.

[B17-behavsci-15-01473] Delgadillo J., Branson A., Kellett S., Myles-Hooton P., Hardy G. E., Shafran R. (2020). Therapist personality traits as predictors of psychological treatment outcomes. Psychotherapy Research.

[B18-behavsci-15-01473] Eysenck H. J. (1970). The structure of human personality.

[B20-behavsci-15-01473] Fabrigar L. R., Wegener D. T. (2012). Exploratory factor analysis.

[B19-behavsci-15-01473] Fabrigar L. R., Wegener D. T., MacCallum R. C., Strahan E. J. (1999). Evaluating the use of exploratory factor analysis in psychological research. Psychological Methods.

[B21-behavsci-15-01473] Field A. (2009). Exploratory factor analysis. Discovering statistics using SPSS.

[B22-behavsci-15-01473] Field A. P. (2018). Discovering statistics using IBM SPSS Statistics.

[B23-behavsci-15-01473] Fraenkel J. R., Wallen N. E. (2006). How to design and evaluate research in education.

[B24-behavsci-15-01473] Friedman H. S., Schustack M. W. (2003). Personality: Classic theories and modern research.

[B25-behavsci-15-01473] Granello D. H., Young M. E. (2019). Counseling today: Foundations of professional identity.

[B26-behavsci-15-01473] Holtzman R. F., Raskin M. S. (1989). Why field placements fail. The Clinical Supervisor.

[B27-behavsci-15-01473] Hua J., Zhou Y.-X. (2023). Personality assessment usage and mental health among Chinese adolescents: A sequential mediation model of the Barnum effect and ego identity. Frontiers in Psychology.

[B28-behavsci-15-01473] Hutcheson G., Sofroniou N. (1999). The multivariate social scientist: Introductory statistics using generalized linear models.

[B29-behavsci-15-01473] Jeanfreau M., Noguchi K., Mong M. D., Stadthagen H. (2018). Financial infidelity in couple relationships. Journal of Financial Therapy.

[B30-behavsci-15-01473] Jung C. G., Baynes H. G., Hull R. F. C. (1974). Psychological types.

[B31-behavsci-15-01473] Kamphuis J. H., Finn S. E. (2018). Therapeutic assessment in personality disorders: Toward the restoration of epistemic trust. Journal of Personality Assessment.

[B32-behavsci-15-01473] Koo T. K., Li M. Y. (2016). A guideline of selecting and reporting intraclass correlation coefficients for reliability research. Journal of Chiropractic Medicine.

[B33-behavsci-15-01473] Lampis J., Cataudella S., Busonera A., Carta S. (2018). Personality similarity and romantic relationship adjustment during the couple life cycle. The Family Journal.

[B34-behavsci-15-01473] Lewis A. J., Locke V., Heritage B., Seddon S. (2022). Trainee therapist personality and the rating of cognitive behavioural and dynamic interpersonal therapy processes. Clinical Psychology & Psychotherapy.

[B35-behavsci-15-01473] MacCallum R. C., Widaman K. F., Zhang S., Hong S. (1999). Sample size in factor analysis. Psychological Methods.

[B36-behavsci-15-01473] Myers I. B., McCaulley M. H., Quenk N. L., Hammer A. L. (2003). MBTI manual: A guide to the development and use of the Myers-Briggs Type Indicator.

[B37-behavsci-15-01473] Newgent R., Parr P., Newman I., Wiggins K. (2017). The Riso-Hudson enneagram type indicator: Estimates of reliability and validity. Measurement and Evaluation in Counseling and Development.

[B38-behavsci-15-01473] Normandin L., Weiner A., Ensink K. (2023). An integrated developmental approach to personality disorders in adolescence: Expanding Kernberg’s object relations theory. American Journal of Psychotherapy.

[B39-behavsci-15-01473] Pearman R. R., Albritton S. C. (1997). I’m not crazy, I’m just not you: The real meaning of the 16 personality types.

[B40-behavsci-15-01473] Perinetti G. (2018). StaTips part IV: Selection, interpretation and reporting of the intraclass correlation coefficient. South European Journal of Orthodontics and Dentofacial Research.

[B41-behavsci-15-01473] Piedmont R. L. (2006). The revised NEO personality inventory (NEO PI-R). Counselor’s guide to clinical, personality, and behavioral assessment.

[B42-behavsci-15-01473] Rath T., Clifton D. O. (2017). Finding your strengths: An introduction to StrengthsFinder 2.0.

[B43-behavsci-15-01473] Rosenblad S. R. (2014). Development and psychometric evaluation of an instrument to identify personality traits in adults. *(Publication No. 3580938)*. Doctoral dissertation.

[B44-behavsci-15-01473] Samuel D. B., Bucher M. A., Suzuki T. (2018). A preliminary probe of personality predicting psychotherapy outcomes: Perspectives from therapists and their clients. Psychopathology.

[B45-behavsci-15-01473] Sheperis C. J., Drummond R. J., Jones K. D. (2020). Assessment procedures for counselors and helping professionals.

[B46-behavsci-15-01473] Sowumni O. A. (2022). Job satisfaction, personality traits, and its impact on motivation among mental health workers. South African Journal of Psychiatry.

[B47-behavsci-15-01473] Sperry L., Carlson J. (2000). Couples therapy with a personality-disordered couple. The Family Journal.

[B48-behavsci-15-01473] Stevens J. (2009). Applied multivariate statistics for the social sciences.

[B49-behavsci-15-01473] Tabachnick B. G., Fidell L. S. (2019). Using multivariate statistics.

[B50-behavsci-15-01473] Watts R. E. (2012). Adlerian counseling: A practitioner’s approach.

[B51-behavsci-15-01473] Widiger T. A., Sellbom M., Chmielewski M., Clark L. A., DeYoung C. G., Kotov R., Krueger R. F., Lynam D. R., Miller J. D., Mullins-Sweatt S., Samuel D. B., South S. C., Tackett J. L., Thomas K. M., Watson D., Wright A. G. C. (2019). Personality in a hierarchical model of psychopathology. Clinical Psychological Science.

